# Radiation-induced cranial meningiomas are associated with earlier relapse: a propensity score–matched cohort study

**DOI:** 10.1007/s11060-026-05809-8

**Published:** 2026-09-22

**Authors:** Leon Schmidt, Dragan Jankovic, Santhosh Thavarajasingam, Reem Fakak, Michael Kosterhon, Alice Dauth, Marc Brockmann, Clemens Sommer, Florian Ringel, Darius Kalasauskas

**Affiliations:** 1 Department of Neurosurgery, University Medical Center Mainz, Mainz, Germany; 2 Department of Neuroradiology, University Medical Center Mainz, Mainz, Germany; 3 Department of Neurosurgery, LMU University Hospital, LMU Medizin, Ludwig-Maximilains-Universität München, Munich, Germany; 4 Department of Neurology, Telemark Hospital Trust, Skien, Norway; 5 Department of Neurosurgery, University Hospital Schleswig-Holstein, Luebeck, Germany; 6 Institute of Neuropathology, University Medical Center Mainz, Mainz, Germany

**Keywords:** Radiation-induced meningioma, Meningioma recurrence, Propensity score matching, Progression-free survival, Extent of resection

## Abstract

**Background:**

Intracranial meningiomas may be induced after radiation exposure, for example following radiotherapy for CNS malignancies. However, data on their clinical characteristics and risk factors for recurrence remain limited. This study aimed to evaluate clinical features and independent predictors of progression-free survival in radiation-induced meningiomas compared to sporadic tumors.

**Methods:**

All cases of radiation-induced (RI) intracranial meningiomas that underwent tumor resection at our institution between 2007 and 2023 were identified. Patients were matched to non-radiation-induced controls by propensity score using age, WHO grade, and Simpson grade as covariates (1:5 ratio). Demographics, tumor characteristics, and outcomes were compared. Survival was analyzed using Kaplan-Meier curves and Cox regression.

**Results:**

A total of 39 RI meningioma cases were matched to 195 non-exposed controls. After matching, there were no significant differences in age, WHO grade, or Simpson grade between groups, confirming adequate baseline balance. Prior cranial surgery was more frequent in the RI group (38.5% vs. 4.1%; *p* < 0.001), and adjuvant radiotherapy was more commonly administered (33.3% vs. 11.3%; *p* < 0.001). Relapse rate was significantly higher in RI tumors (28% vs. 17%; *p* = 0.001). In multivariate Cox regression, RI tumor status (HR 2.1; 95% CI 1.02–434; *p* = 0.04) and complete resection (HR 0.29; 95% CI 0.15–0.54; *p* < 0.001) were independent predictors of progression-free survival.

**Conclusion:**

Radiation-induced meningioma status was associated with shorter progression-free survival following propensity score matching and multivariable adjustment. Complete resection was associated with improved PFS, supporting maximal safe resection when clinically feasible. However, residual measured and unmeasured confounding cannot be excluded, and the present findings should not be interpreted as evidence of intrinsically more aggressive tumor biology.

**Supplementary Information:**

The online version contains supplementary material available at https://doi.org/10.1007/s11060-026-05809-8.

## Introduction

Meningiomas are the most common primary intracranial neoplasm, accounting for up to 40% of all primary central nervous system (CNS) tumors, with a population-based incidence of approximately 8–9 per 100,000 person-years [1, 2]. Detection rates have risen further with the increasing use of cranial imaging, and the clinical course ranges from incidentally discovered, indolent lesions to aggressive tumors accompanied by perifocal edema that require surgical intervention [3]. Established determinants of recurrence after resection include WHO histological grade, extent of resection as defined by the Simpson grading system, tumor location, and patient age [4–6]. The majority of meningiomas arise sporadically, and their etiology remains largely unclear; however, several risk factors have been identified [7, 8]. Female sex hormones are thought to play a role, given the higher incidence in women and the frequent expression of progesterone receptors in tumor tissue [9]. Notably, long-term use of synthetic progestogens, particularly cyproterone acetate, but also chlormadinone acetate and nomegestrol acetate, has been consistently associated with an increased risk of meningioma development and growth, with several case series and pharmacoepidemiological studies demonstrating tumor regression after discontinuation [10]. Prior exposure to ionizing radiation represents another well-established, though less common, risk factor [11]. 

A smaller, clinically distinct subset of meningiomas arises after prior exposure to ionizing radiation, so-called radiation-induced (RI) meningiomas [12]. These tumors most commonly develop decades after cranial irradiation administered for childhood malignancies, pituitary disease, or other CNS tumors, with a latency period between exposure and diagnosis typically ranging from 15 to 35 years [13]. As survival after radiotherapy-treated malignancies continues to improve, the population at risk for developing RI meningiomas later in life is expected to grow, increasing the clinical relevance of this entity [14]. Several descriptive series have reported that RI meningiomas present at a younger age, more frequently as multiple synchronous lesions, with a higher proportion of atypical (WHO grade 2) histology, and with a more aggressive clinical course and higher recurrence rates than sporadic tumors [11, 15, 16]. The biological basis for this apparent aggressiveness remains incompletely understood, though radiation-induced chromosomal instability and molecular alterations distinct from those of sporadic meningiomas have been proposed as contributing mechanisms [16]. Despite these observations, most existing literature on RI meningiomas derives from unmatched, single-arm descriptive cohorts or from comparisons with historical sporadic populations that differ systematically in age, WHO grade distribution, and extent of resection, all of which are independently associated with recurrence [12, 17]. Without adequate control for these confounders, it remains unclear whether the reportedly worse outcomes of RI meningiomas reflect an intrinsically more aggressive tumor biology, or whether they are, at least in part, attributable to differences in baseline patient and tumor characteristics between exposed and unexposed populations. Propensity score matching offers a way to address this limitation by constructing a comparison group with improved balance on selected prognostic factors, thereby reducing, although not eliminating, potential confounding.

To date, however, no study has applied this approach to directly compare recurrence risk between RI and sporadic meningiomas. This study aims to characterize the clinical, radiological, and histopathological features of RI meningiomas and to identify independent predictors of recurrence matched by propensity score to sporadic controls on age, WHO grade, and Simpson grade.

## Materials and methods

All cases of radiation-induced (RI) intracranial meningioma that underwent tumor resection at our institution between 2007 and 2023 were identified. RI meningioma was defined according to Cahan’s criteria: (1) the tumor arose within a previously irradiated field (2), a sufficient latency period of at least 5 years had elapsed between irradiation and the diagnosis of meningioma (3), the histological diagnosis of meningioma was confirmed, and (4) the tumor was not present at the time of initial irradiation [18]. 

Patients were ≥ 18 years of age and have undergone resection of at least one intracranial meningioma. For patients with multiple synchronous RI lesions, the lesion prompting surgical intervention was designated as the index tumor for outcome analysis. This study followed the STROBE guidelines for observational cohort studies [19]. 

Demographics and tumor-related characteristics were retrospectively evaluated using institutional medical records, radiological reports, and pathology reports. Preoperative functional status was assessed using the Karnofsky Performance Status (KPS), and comorbidity burden was quantified using the Charlson Comorbidity Index (CCI) [20, 21]. 

Histopathological diagnoses and grades were obtained from the original pathology reports. Historical specimens were not systematically rereviewed; instead, the available pathological diagnoses and grading information were retrospectively mapped to the 2021 WHO classification of CNS tumors where sufficient information was available [22]. Extent of resection was defined using the Simpson grading system, with complete resection defined as Simpson grade I or II [23]. 

Postoperative surveillance followed institutional protocol, with imaging performed at 3, 6, and 12 months and annually thereafter. Radiographic recurrence or progression was adjudicated by the treating neurosurgical team. Following complete resection (Simpson grade I–II), recurrence was defined as the appearance of a new enhancing lesion at or adjacent to the surgical site on follow-up MRI that was considered consistent with recurrent meningioma. Following incomplete resection (Simpson grade III–IV), progression was defined as radiographically documented growth of residual tumor on serial follow-up MRI. Equivocal postoperative enhancement or minor interval changes were not classified as recurrence or progression unless confirmed on subsequent imaging. Treatment decisions were made during interdisciplinary institutional tumor board meetings.

Progression-free survival (PFS), measured in months, was defined as the time from surgery to the first radiographically documented recurrence or progression. Patients without documented recurrence or progression were censored at the date of their last available radiographic follow-up. Patients who died without documented recurrence or progression were censored at the date of death. Patients without evaluable postoperative imaging follow-up were excluded from the PFS analysis.

### Propensity score matching

To reduce selection bias, propensity scores were calculated using a logistic regression model incorporating age, WHO grade, and Simpson grade as covariates. Age, WHO grade, and Simpson grade were selected a priori because of their clinical relevance to meningioma recurrence and PFS, with the aim of improving comparability between groups with respect to age, tumor grade, and extent of resection. Although WHO grade and Simpson grade are not strictly pretreatment baseline characteristics, they were included because of their prognostic relevance to the primary outcome. Given the limited number of RI cases, the propensity score model was deliberately restricted to these key prognostic factors rather than including a larger number of covariates. From an initial pool of 817 eligible historical non-RI meningioma cases, controls were matched to RI cases using nearest-neighbor matching at a 1:5 ratio (one exposed patient to five controls), without replacement, using a caliper width of 0.2 SD of the logit of the propensity score and the MatchIt package in R (RRID: SCR_025618) [24]. Post-matching covariate balance was assessed using standardized mean differences (SMD). An absolute SMD < 0.10 was considered indicative of adequate covariate balance. (Supplementary Figure. 1).

### Statistical analysis

Categorical variables are presented as absolute and relative frequencies and were compared using the χ²-test or Fisher’s exact test, as appropriate. Normality of continuous variables was assessed using the Shapiro-Wilk test; results are presented as mean ± SD or median (interquartile range, IQR), and groups were compared using the Wilcoxon rank-sum test. Survival was analyzed using the Kaplan-Meier method with log-rank testing for univariable comparisons. The median follow-up time was estimated using the reverse Kaplan-Meier method, with the event indicator reversed to account for censoring [25]. No imputation of missing data was performed. Propensity score matching was restricted to patients with complete data for all variables included in the propensity score model (age, WHO grade, and Simpson grade). Multivariable Cox regression was performed as a complete-case analysis, including only patients with complete data for all covariates included in the final model and available PFS information. To reduce the risk of overfitting given the limited number of progression events, a parsimonious multivariable Cox proportional hazards regression model was fitted including RI status, complete resection, and surgically complex localization as covariates. The proportional hazards assumption was assessed using Schoenfeld residuals, the proportional hazards assumption was not violated. A two-sided p-value < 0.05 was considered statistically significant; no correction for multiple comparisons was applied given the exploratory nature of secondary analyses. All analyses were performed in R version 4.5.3 [26]. 

### Ethics

According to the local laws of Rhineland-Palatinate, Germany, no formal ethics approval or informed consent was required for this retrospective analysis. All patient data were de-identified prior to analysis.

## Results

### Study population and matching

Between 2007 and 2023, 39 cases of RI meningioma underwent surgical resection at our institution. From an initial pool of 817 eligible historical non-RI cases, 195 controls were selected using 1:5 propensity score matching. After matching, covariate balance was substantially improved. The post-matching SMD for age was 0.050. Absolute SMDs were 0.084, 0.085, and < 0.001 for WHO grades 1–3, respectively, and 0.082, 0.175, 0.026, and 0.101 for Simpson grades 1–4, respectively. Although most covariate levels demonstrated good balance, some residual imbalance remained for Simpson grade (Supplementary Figure. 1). Following propensity score matching, RI patients had a significantly higher comorbidity burden (median CCI 2 [IQR 0-3.25] vs. 0 [IQR 0–1]; *p* < 0.001) and had undergone prior cranial surgery more frequently (38.5% vs. 4.1%; *p* < 0.001). Preoperative KPS was modestly lower in RI patients (80 [IQR 70–90] vs. 80 [IQR 80–90]; *p* = 0.01). Sex distribution did not differ significantly between groups (69% female in RI vs. 77% in controls; *p* = 0.42). There were no significant differences in tumor diameter (27.3 [22.7–45.7] vs. 26 [17–44] mm; *p* = 0.75), tumor localization (*p* = 0.21), or proportion of surgically complex cases (25.6% vs. 24.6%; *p* = 1.0). Baseline characteristics are presented in Table 1.

**Table 1:** Baseline variables compared between radiation-induced and non-radiation-induced meningioma cohorts after propensity score matching

Variable	Radiation-induced (*N* = 39)	Control (*N* = 195)	*p*-value
Age (median, IQR) [years]	44 (35–49)	44 (39–52)	0.2
Sex (female)	27(69%)	151(77%)	0.42
Charlson Comorbidity Index (median, IQR)	2 (0–3.25)	0 (0–1)	< 0.001
KPS (preoperative, median, IQR)	80 (70–90)	80 (80–90)	0.01
Previous cranial surgery	15 (38.5%)	8 (4.1%)	< 0.001
Tumor diameter (median, IQR) [mm]	27.3 (22.7–45.7)	26 (17–44)	0.75
Localization			0.21
Convexity	18 (46.2%)	62 (31.8%)	
Falx	7 (17.9%)	28 (14.4%)	
Fronto-basal	3 (7.7%)	34 (17.4%)	
Sphenoid wing	4 (10.3%)	31 (15.9%)	
Petroclival	2 (5.1%)	9 (4.6%)	
Other	5 (12.8%)	31 (15.9%)	

IQR = interquartile range; CCI = Charlson Comorbidity Index; KPS = Karnofsky Performance Score. Comparison by Wilcoxon rank-sum test, χ²-test, or Fisher’s exact test as appropriate

### Surgical and histopathological findings

Complete resection (Simpson grade I-II) was achieved in 71.8% of RI cases and 76.4% of controls (*p* = 0.68). WHO grade 1 tumors were present in 69.2% of RI patients and 77.4% of controls (*p* = 0.39), with transitional histology the most common subtype in both groups. Adjuvant radiotherapy was administered more frequently in RI patients (33.3% vs. 11.3%; *p* < 0.001). Postoperative KPS at 6-month and final follow-up did not differ significantly between groups. Details are provided in Table 2.

**Table 2:** Surgical, histopathological, and follow-up characteristics by cohort

Variable	Radiation-induced (*N* = 39)	Control (*N* = 195)	*p*-value
Extent of resection (complete)	28 (71.8%)	149 (76.4%)	0.68
Simpson class 1	21 (53.8%)	85 (43.6%)	0.19
Simpson class 2	7 (17.9%)	64 (32.8%)	
Simpson class 3	6 (15.4%)	33 (16.9%)	
Simpson class 4	5 (12.8%)	13 (6.7%)	
Vessel manipulation	7 (17.9%)	33 (16.9%)	0.97
Cranial nerve manipulation	6 (15.4%)	30 (15.4%)	0.87
WHO grade 1	27 (69.2%)	151 (77.4%)	0.39
WHO grade 2	12 (30.8%)	42 (21.5%)	
WHO grade 3	0 (0%)	2 (1.0%)	
Adjuvant radiotherapy	13 (33.3%)	22 (11.3%)	< 0.001
KPS at 6-month follow-up	90 (70–90)	90 (80–90)	0.6
KPS at last follow-up	90 (80–100)	90 (80–90)	0.7

### Univariable survival analysis

**Fig. 1:**
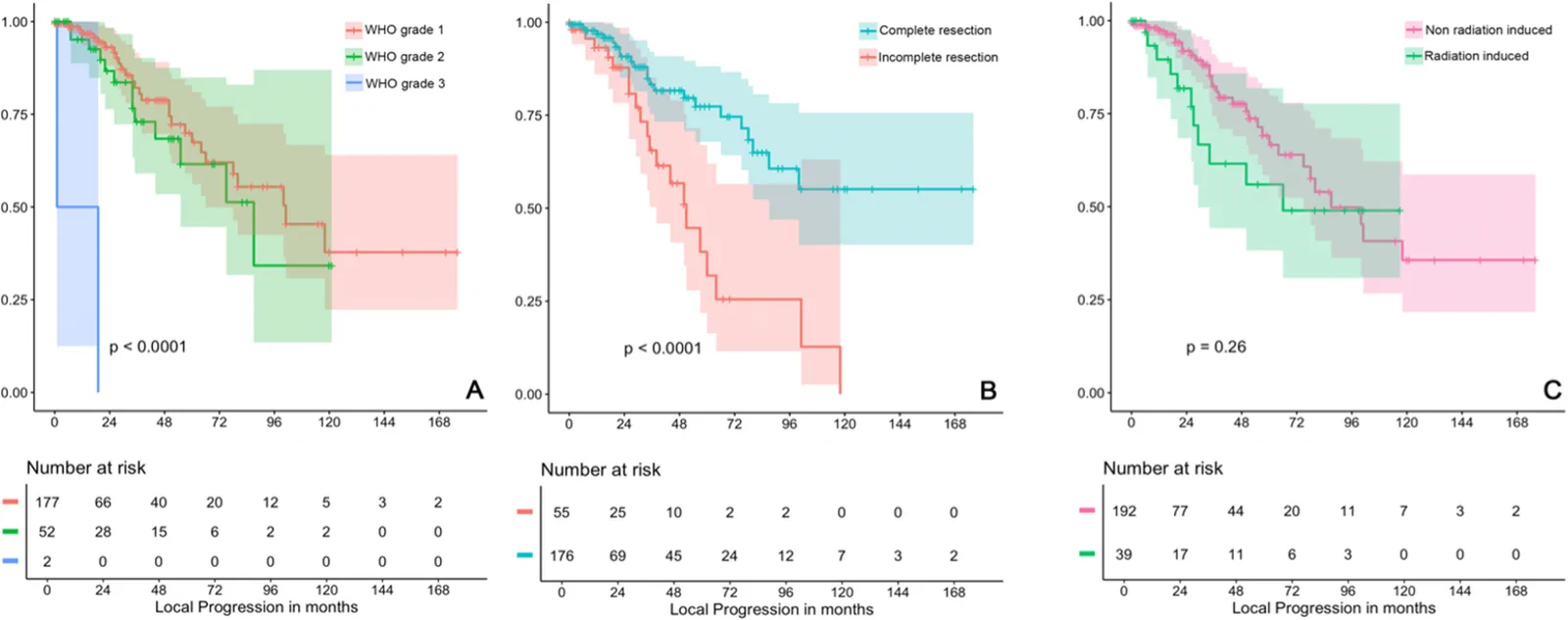
Progression-free survival (PFS) in the overall matched cohort. (**a**) PFS stratified by WHO grade. (**b**) PFS stratified by extent of resection. (**c**) PFS stratified by radiation induction. Survival differences were evaluated using the log-rank test

Median follow-up estimated using the reverse Kaplan–Meier method was 20 months (IQR 4–55) for the overall cohort, 41 months (IQR 6–84) for RI patients, and 18 months (IQR 4–51) for controls. A total of 44 relapse events were observed (11/39 in RI patients vs. 33/195 in controls; *p* = 0.001). Median PFS was 66 months in RI patients versus 88 months in controls. In univariable analysis, WHO grade and complete resection were significant predictors of PFS (each *p* < 0.001); Kaplan-Meier curves stratified by radiation exposure, WHO grade, and extent of resection are shown in Fig. 1.

### Multivariable survival analysis

Two-hundred and thirty complete cases with 43 events were available for multivariable analysis. The multivariable Cox proportional hazards model was statistically significant (likelihood-ratio test, χ²=17.65, *p* < 0.001) and showed a concordance index of 0.635 (SE = 0.054). On multivariable Cox regression adjusting for complete resection and surgically complex localization, RI tumor status (HR 2.1; 95% CI 1.02–434; *p* = 0.04) and complete resection (HR 0.29; 95% CI 0.15–0.54; *p* < 0.001) were the only independent predictors of PFS, surgically complex location did not achieve a significant association (HR1.49; 95% CI 0.8–2.79; *p* = 0.2). The proportional hazards assumption was not violated for any of the covariates (RI status: *p* = 0.091; complete resection: *p* = 0.101; surgically complex localization: *p* = 0.504), with no evidence of a global violation (*p* = 0.207).

## Discussion

In our propensity score–matched cohort, radiation-induced meningioma status was associated with earlier tumor recurrence compared with sporadic meningiomas, and this association persisted after multivariable adjustment. Complete surgical resection was significantly associated with improved PFS.

Our findings are consistent with prior comparative studies suggesting a higher recurrence propensity in RI meningiomas. Shah et al. reported a markedly higher recurrence rate in RI compared with sporadic meningiomas in an unmatched single-center case-control cohort (50% vs. 5%), while Kim et al. similarly described a less favorable clinical course in RI meningiomas [16, 17]. However, previous comparisons were based largely on unmatched RI and sporadic cohorts, leaving greater potential for differences in prognostically relevant characteristics to influence the observed outcomes. By applying propensity score matching for age, WHO grade, and Simpson grade, the present study improves comparability between RI and sporadic meningiomas with respect to these selected prognostic factors. Our findings therefore provide a methodologically strengthened confirmation and refinement of the previously reported association between RI status and recurrence rather than establishing this association as a novel observation. To our knowledge, this is among the first studies to apply propensity score matching to directly compare recurrence outcomes between RI and sporadic meningiomas. Nevertheless, matching did not eliminate all clinically relevant differences between the groups, and residual confounding should be considered when interpreting the observed association.

RI meningioma patients in our cohort presented with a higher comorbidity burden and more frequent prior cranial surgery. Although propensity score matching improved comparability with respect to the selected matching variables, clinically relevant differences between the groups remained. The higher proportion of prior cranial surgery in RI patients likely reflects previous interventions related to the underlying condition for which cranial irradiation was initially administered. Preoperative KPS was lower in RI patients, whereas no significant differences in tumor localization or surgical complexity were observed. Rates of complete resection were comparable between RI and non-RI groups (71.8% vs. 76.4%). However, as Simpson grade was included as a covariate in the propensity score matching, this similarity is expected by study design and should not be interpreted as evidence of comparable resectability between RI and sporadic meningiomas. Notably, complete resection remained independently associated with improved PFS in the multivariable analysis, emphasizing the prognostic relevance of extent of resection in this cohort.

Postoperative functional performance, assessed at 6 months and final follow-up using the KPS, did not differ significantly between groups. However, procedure-specific neurological morbidity and other surgical complications were not systematically evaluated; therefore, the absence of a difference in postoperative KPS should not be interpreted as evidence of equivalent surgical morbidity between RI and sporadic meningioma patients. Adjuvant radiotherapy was more frequently employed in RI patients (33.3% vs. 11.3%), potentially reflecting differences in tumor grade and other clinical considerations.

The observed association between RI status and recurrence risk persisted after multivariable adjustment, suggesting that differences in outcome may not be fully explained by WHO grade or extent of resection alone. However, given the residual clinical differences between the groups, including comorbidity burden, preoperative KPS, and prior cranial surgery, this association should not be interpreted as an isolated effect of radiation-induced status. Residual measured and unmeasured confounding may have contributed to the observed difference in PFS. Furthermore, molecular data were not systematically available in our cohort, precluding conclusions regarding whether the observed clinical differences reflect intrinsically distinct tumor biology. Previous studies have suggested that radiation-induced DNA damage, chromosomal instability, and specific molecular alterations may contribute to distinct biological features of RI meningiomas compared with sporadic meningiomas [27, 28]. Further molecular studies are needed to determine whether such biological differences contribute to the observed clinical outcomes.

### Limitations

This study has several limitations inherent to its retrospective, single-center design. The relatively small number of RI cases (*n* = 39) limits statistical power for subgroup analyses. Although a parsimonious multivariable Cox regression model was used to reduce the risk of overfitting, the relatively limited number of relapse events may still affect the precision and stability of the effect estimates. Therefore, the magnitude of the observed associations should be interpreted with caution and requires validation in larger cohorts with a greater number of events. Median follow-up of 20 months is relatively short for meningioma recurrence assessment, given that late recurrences beyond 5 years are well described. In addition, follow-up duration differed between groups, with longer follow-up in RI patients than in controls. Given the retrospective study design, differences in surveillance duration and imaging frequency between RI and sporadic meningioma patients cannot be excluded and may have influenced the probability and timing of radiographic detection of recurrence or progression. Molecular data were not systematically available. In addition, historical pathological specimens were not systematically rereviewed according to the 2021 WHO classification, and retrospective mapping of the original histopathological diagnoses and grading information may have introduced some degree of grade misclassification, particularly in older cases. Furthermore, propensity score matching did not eliminate all clinically relevant differences between the groups. RI patients had a higher comorbidity burden, lower preoperative KPS, and more frequently underwent prior cranial surgery. As these variables were not included in the propensity score model, residual confounding cannot be excluded. Consequently, the observed association between RI status and PFS should not be interpreted as an isolated effect of radiation-induced status and may, in part, reflect residual measured or unmeasured differences between the groups. Despite these limitations, propensity score matching represents a methodological strength allowing for more valid group comparisons than unmatched historical controls.

## Conclusion

Radiation-induced meningioma was associated with earlier tumor recurrence compared with propensity score-matched sporadic meningiomas, and this association persisted after multivariable adjustment. Complete surgical resection was associated with improved PFS, supporting maximal safe resection when clinically feasible. These findings should be interpreted in the context of the retrospective study design and potential residual confounding. Long-term surveillance remains important in patients with RI meningiomas.

## Supplementary Information

Below is the link to the electronic supplementary material.

Supplementary Material 1

## Data Availability

No datasets were generated or analysed during the current study.
