# Supplementary material for: Radiation-induced cranial meningiomas are associated with earlier relapse: a propensity score–matched cohort study
Source: J Neurooncol. 2026 Sep 22;179(3):97. doi: 10.1007/s11060-026-05809-8 (PMC13597638; doi:10.1007/s11060-026-05809-8)
Supplement: Supplementary file 1 [file 11060_2026_5809_MOESM1_ESM.docx]

**Supplementary**

**Figure 1**. **Covariate balance before and after propensity score matching.** Absolute standardized mean differences (SMDs) are shown for the covariates included in the propensity score model before and after matching. The dashed vertical line indicates an absolute SMD of 0.10, the prespecified threshold for acceptable covariate balance. Propensity score matching substantially improved covariate balance, although some residual imbalance remained for Simpson grade.
